# Can the success of structured therapy for giggle incontinence be predicted?

**DOI:** 10.1590/S1677-5538.IBJU.2014.0560

**Published:** 2016

**Authors:** Onur Telli, Nurullah Hamidi, Aytac Kayis, Evren Suer, Tarkan Soygur, Berk Burgu

**Affiliations:** 1 Department of Paediatric Urology, School of Medicine, Ankara University, Ankara, Turkey; 2 Department of Urology, School of Medicine, Ankara University, Ankara, Turkey

**Keywords:** Urinary Incontinence, Methylphenidate, Laughter

## Abstract

**Introduction:**

To evaluate possible factors that can guide the clinician to predict potential cases refractoriness to medical treatment for giggle incontinence (GI) and to examine the effectiveness of different treatment modalities.

**Material and methods:**

The data of 48 children referred to pediatric urology outpatient clinic between 2000 and 2013 diagnosed as GI were reviewed. Mean age, follow-up, GI frequency, associated symptoms, medical and family history were noted. Incontinence frequency differed between several per day to less than once weekly. Children were evaluated with uroflowmetry-electromyography and post-void residual urine. Clinical success was characterized as a full or partial response, or nonresponse as defined by the International Children's Continence Society. Univariate analysis was used to find potential factors including age, sex, familial history, GI frequency, treatment modality and dysfunctional voiding to predict children who would possibly not respond to treatment.

**Results:**

Mean age of the patients was 8.4 years (range 5 to 16). Mean follow-up time and mean duration of asymptomatic period were noted as 6.7±1.4 years and 14.2±2.3 months respectively. While 12 patients were treated with only behavioral urotherapy (Group-1), 11 patients were treated with alpha-adrenergic blockers and behavioral urotherapy (Group-2) and 18 patients with methylphenidate and behavioral urotherapy (Group-3). Giggle incontinence was refractory to eight children in-group 1; six children in-group 2 and eight children in-group 3. Daily GI frequency and dysfunctional voiding diagnosed on uroflowmetry-EMG were found as outstanding predictive factors for resistance to treatment modalities.

**Conclusions:**

A variety of therapies for GI have more than 50% failure rate and a standard treatment for GI has not been established. The use of medications to treat these patients would not be recommended, as they appear to add no benefit to symptoms and may introduce severe adverse effects.

## INTRODUCTION

Giggle incontinence (GI) or enuresis risoria (ER) is characterized by complete or involuntary bladder emptying occurring only with giggling or laughing while awake with normal bladder function at all other times (1). GI is different from stress incontinence and overactive bladder because it falls under the urge type: laughter suddenly induces acute, uncontrollable urgency immediately followed by micturition that cannot be stopped until the bladder is completely empty. The pathophysiology of GI has been poorly understood and although giggle incontinence is thought to be fairly common, various pharmacological and behavioral therapies have limited success and it is generally considered that there is no known specific treatment for the disorder (2). Currently, available treatment strategies include standard urotherapy, bladder and pelvic floor retraining and methylphenidate (MPH) (3–5). For patients in whom the MPH failed or was not tolerated, treatment focuses on behavioral modifications such as frequent bladder emptying with alpha-blocker therapy (6).

The present study examined possible factors that can guide clinician to predict cases refractory to treatment of GI. The secondary outcome of the present study was to compare GI patients response to treatment modalities of urotherapy, alpha-adrenergic blockers and MPH.

## MATERIALS AND METHODS

After approved by the institutional review board, the data of 48 children referred to pediatric urology outpatient clinic between 2000 and 2013 diagnosed as giggle incontinence were retrospectively investigated. The diagnosis of giggle incontinence was made by definition of the International Children's Continence Society (1). Detailed history and physical examination, including neurologic examination, was performed in all children. Children were also personally interviewed on voiding and defecation habits, and giggle incontinence. Data were collected and analyzed on GI frequency, age, gender, voiding patterns, associated symptoms, medical and family history. Uroflowmetry with pelvic floor EMG was used to evaluate dysfunctional voiding and urinary ultrasound measurement of post-void residual urine. Children with other types of enuresis, urge incontinence, history of urinary tract infection or neurogenic voiding dysfunction and those with known structural urinary tract obstruction or complex urinary tract malformations were excluded from study.

Patients were grouped into 3 groups. Group-1 consisted of children who were treated with only behavioral urotherapy, Group-2 consisted of children who were treated with behavioral urotherapy and alpha-adrenergic receptor antagonist (doxazosin) and Group-3 consisted of children who were treated with behavioral urotherapy and MPH.

Behavioral urotherapy consisted of hydration; maintaining an empty bladder including timed and double voiding and avoiding bladder stimulants (coffee, tea, etc.); toilet training and bowel management. In Group-2 patients were treated with doxazosin, 0.5mg dose per day and in Group-3 patients were treated with MPH, 5mg dose per day, for at least a 6-month period (3, 6, 7). They had all been informed of the prescription and adverse effects.

Clinical success was characterized as a full response—100% decrease in symptoms or less than 1 occurrence monthly, a partial response—50% to 89% decrease, or a nonresponse—0% to 49% decrease, as defined by the International Children's Continence Society (1).

All statistical analysis was performed using SPSS ver.16.5 (Statistical Package for Social Sciences for Windows 16.5 Inc., Chicago, Il, USA). All values were expressed as mean±standard error (SEM). Between groups, analyses were performed using chi square test. Univariate Cox regression analysis was performed to evaluate the predictors for resistance to treatment modalities and the 95% CI was selected, and p value of 0.05 was set for statistically significance.

## RESULTS

A total of 48 patients presented with giggle incontinence at our institution between 2000 and 2013. Of these, 7 patients were excluded from study due to missing data. The remaining 41 patients, 32 females and 9 males with a mean age of 8.4 years (range 5 to 16 years) were identified. Patient characteristics are summarized in Table-1. There were 12 patients (male/female: 10/2) with a mean age of 8.2 in Group-1, 11 patients (male/female: 7/4) with a mean age of 8.3 in Group-2 and 18 children (male/female: 15/3) with a mean age of 8.6 in Group-3. Mean follow-up time and mean duration of asymptomatic period were noted as 6.7±1.4 years and 14.2±2.3 months, respectively. There were no differences in age, sex, GI frequency, and family history between groups (p>0.05). On uroflowmetry-EMG, 4 (33%) patients in Group-1, 4 (36%) patients in Group-2 and 6 (33%) in Group-3 were found to have dysfunctional voiding.

**Table 1:** Characteristics of patients with GI.

		Group 1 (behavioral urotherapy)	Group 2 (alpha blocker+behavioral urotherapy)	Group 3 (MPH+behavioral urotherapy)
No. Patients		12	11	18
Age in years		8.2±2.0	8.3±1.6	8.6±1.6
Gender (M/F)		10/2	7/4	15/3
**Frequency GI**				
	Daily	3 (25%)	3 (28%)	4 (22%)
	Weekly	4 (33%)	4 (36%)	7 (39%)
	Less than weekly	5 (42%)	4 (36%)	7 (39%)
	Family history	5 (42%)	5 (39%)	7 (39%)
	Full response	0 (0%)	1 (9%)	6 (33%)
	Partial response	4 (33%)	4 (36%)	4 (22%)
	Refractor to treatment (non-response)	8 (66%)	6 (54%)	8 (44%)

In Group-1, 8 children of 12 patients; in Group-2, 6 of 11 patients and 8 children of 18 patients in Group-3 reported 0% to 49% decrease after at least 6 months of treatment and therefore the outcome of treatment was considered refractor to treatment. Univariate analysis was used to find potential factors including age, sex, familial history, GI frequency and dysfunctional voiding (uroflowmetry-EMG) for predictive factors of treatment resistance. Daily GI frequency (OR; 1.910, 95% CI 1.338-2.845; p=0.041) and dysfunctional voiding, (OR; 2.128, 95% CI 1.244-3.196; p=0.012) were statistically significantly associated with resistance to medical treatment in univariate analyses (Table-2).

**Table 2:** Univariate logistic regression analysis of predictors for resistance to medical treatment of GI.

Variable		OR	95%Cl	P value
**Age**		1.008	0.934-1.125	0.869
**Sex**				
	Male	1.000	0.952-2.146	0.074
	Female	1.838		
**GI Frequency**				
	Less Than weekly	1.000	1.338-2.845	0.041
	Weekly	1.000		
	Daily	1.910		
**Dysfunctional voiding**				
	Negative	1.000	1.244-3.196	0.012
	Positive	2.128		
	Family History	1.594	0.942-1.955	0.561
**Treatment Modality**				
	MPH+BU	1.000	0.836-2.024	0.362
	Alpha blocker+BU	1.000		
	BU	1.322		

**MPH =** methylphenidate; **BU =** behavioral therapy

## DISCUSSION

GI is a rare condition with unknown cause that is different from stress incontinence and overactive bladder. Typically GI patients report incontinence only with laughter. The incidence may be hidden because of affected patients modify their behavior to avoid embarrassments in social life. The pathophysiology of GI has not been understood therefore a specific treatment has not been established and there is no widely accepted treatment of choice.

In the current study, we have determined the predictor factors of refractoriness to treatment modalities for GI and to the best of our knowledge, there is no study evaluating the predictors for resistance to treatment modalities of GI that can guide the clinician to predict potential cases refractory to medical treatment. A second goal of our study was to evaluate the success of behavioral urotherapy, alpha-adrenergic blockers and MPH treatment for GI and we found that similar patient populations of study groups are refractory to treatments.

Present study reports that daily GI frequency and dysfunctional voiding diagnosed on uroflowmetry-EMG were the potential predictors of resistance to treatment modalities. Although GI is supplemented by the absence of other voiding symptoms and usually established on history, similar to our study Richardson et al. (5) reported 40% of the patients had dysfunctional voiding sequel diagnosed with uroflowmetry-EMG. The children with GI improved sphincter tone and muscle recruitment using biofeedback techniques, supplemented behavior modifications and pharmacotherapy or avoided the need for pharmacotherapy when at least 4 sessions were performed. Additionally, Chandra et al. (2) hypothesized that GI might be secondary to detrusor instability since 95% of their patients had concomitant dysfunctional voiding symptoms in contrast to GI etiology as defined by the International Children's Continence Society (1). Despite the majority of past studies GI showed some subjective improvement in lower urinary tract function. Chang et al. (8) reported that MPH is one of the primary treatment related to increasing urethral closure pressure while the relationship between GI and detrusor overactivity was not possible to be established in their study even including urodynamic parameters. Botulinum toxin A injection has also been used for the treatment of GI that might be related to detrusor overactivity (9). Sher and Reinberg showed that GI has a functional relationship to cataplexy and narcolepsy with a common pathophysiological basis since laughter or emotion may trigger changes in muscle tone (10). They have shown that stimulant medication such as MPH may be successful in their 7 pediatric patients. Both GI and narcolepsy are centrally mediated disorders and share a common pathophysiological basis, which is related to receptor-mediated imbalance of cholinergic and monoaminergic systems. Blockage of norepinephrine uptake mediates the anti-cataplectic effect of currently prescribed antidepressants, while blockade of dopamine uptake and/or stimulation of dopamine release mediate the awake-promoting effect of central nerve system stimulants (11). Therefore, MPH, behaving mainly as a dopamine reuptake inhibitor, is a common therapy for GI and narcolepsy. Berry et al. also reported an 80% cure rate of MPH for GI in 12 of 15 patients. In our study, patients treated with MPH reported 44% failure rates (8/18). Our results with MPH were inferior to previous reports that may be related to single day 5mg MPH usage in our study; the drug is typically administered in multiple daily doses in other clinical settings, based on body weight or body surface area due to its short half-life.

In our daily practice, children with urinary incontinence have behavioral modifications such as frequent bladder emptying with alpha-blocker therapy as a treatment option. GI patients have the same alpha-blocker therapy with bladder emptying treatment and similar to the MPH treatment for GI, children may sometimes be presented with resistance to such therapies. Stimulation of the large concentration of alpha-receptors found in the region of the bladder neck and urethra results in increased outlet resistance. Cain and colleagues (6) reported on 55 consecutive children with urinary incontinence and inadequate bladder emptying treated with Doxazosin at a dose of 0.5-2mg/day in addition to other 'standard' therapy. They found an 88% reduction in post-void residual urine volumes, improvement in urinary incontinence in 83% and a reduction in urgency in 70% although convincing evidence is still awaited.

Population studies indicate that a major narcolepsy/cataplexy gene is linked to HLA-DR2, which may be related with the strong positive family history of giggle incontinence (10, 12). A positive family history was reported in a previous study for giggle incontinence in 13% of the patients that it was not found as a predictor factor for refractoriness to treatment of GI in our study (2).

Unfortunately, our study involved a relatively small number of study groups with unreachable lack of information, which is the major limitation of retrospective review. Future multicenter studies encompassing larger sample size incorporating a prospective double-blind placebo control design with longer follow-up periods are needed to evaluate the success of treatment modalities, predictors that affect the success of treatment modalities and relationship between GI and detrusor inability.

## CONCLUSIONS

Despite the retrospective and non-randomized nature, the results of the present study revealed that medical therapy for GI is accompanied with a failure rate of more than 50%, regardless of the association with any kind of drug therapy. Based on the data from the present study, the use of medication in these patients should be avoided because they appear to add no benefit to the patients and may introduce severe adverse effects.
